# A Seven-Year Etiological Study of Adult Bowel Obstruction in Shiraz, Iran

**DOI:** 10.34172/aim.2023.37

**Published:** 2023-05-01

**Authors:** Seyed Ali Hosseini, Mohammed Abdzaid Akool, Amir Hossein Emami Meybodi, Seyed Vahid Hosseini

**Affiliations:** ^1^Student Research Committee, Shiraz University of Medical Sciences, Shiraz, Iran; ^2^Colorectal Research Center, School of Medicine, Shiraz University of Medical Sciences, Shiraz, Iran; ^3^Department of Surgery, Faculty of Medicine, Jabir Ibn Hayyan Medical University, Najaf, Iraq; ^4^Department of Surgery, School of Medicine, Shiraz University of Medical Sciences, Shiraz, Iran

**Keywords:** Colorectal neoplasms, Intestinal obstruction, Iran, Large bowel obstruction, Small bowel obstruction

## Abstract

**Background:**

Bowel obstruction is a disorder in the passage of bowel contents, the etiology of which varies depending on temporal and geographical conditions. This study investigated the etiology of bowel obstruction in a large number of patients at an adult surgery referral center in southern Iran.

**Methods:**

In this cross-sectional study, we reviewed the medical records of all patients admitted to Shahid Faghihi hospital (Shiraz, Iran) between 2014 and 2020 with a diagnosis of small or large bowel obstruction. Patients with missing or obscure data on etiology were excluded. Data was collected on the patients’ age, gender, history of relevant surgeries, comorbidities, cause of obstruction, site/type of obstruction, treatment, intensive care unit (ICU) admission, length of hospital stay, and outcome. Statistical analyses were made using SPSS v. 25.0.

**Results:**

A total of 2781 bowel obstruction patients (61.4% males, 38.6% females) with a median age of 58 (IQR 43-71) years were studied. Most responded to non-surgical treatment (61.3%). While the obstruction was mostly partial (65.5%), 94.4% of patients with complete obstruction required surgery. Small bowel obstruction (SBO) was almost three times more prevalent than large bowel obstruction (LBO). Adhesion bands were the leading cause of SBO (66.77%), while LBO was primarily due to colorectal tumors (33.9%).

**Conclusion:**

The predominant etiology of LBO was colorectal cancer, suggesting that policymakers should improve surveillance programs to detect the condition earlier. Regarding SBO, the leading cause was adhesion bands, indicating the necessity of further efforts to reduce the rate of adhesions following intra-abdominal operations.

## Introduction

 Bowel obstruction is a disorder in the passage of bowel contents through the bowels. The obstruction may be complete or partial, mechanical or paralytic, and in the small or large bowel. Reference books list various etiologies. As an urgent medical condition, the rate of mortality is high if appropriate treatment is not provided promptly.

 Temporal and geographical differences result in different epidemiological features of small bowel obstruction (SBO). Prior to 1900, hernias were the most common cause. However, due to the high rate of elective hernia repair, hernias currently rank third among the causes of SBO.^1^ Currently, intra-abdominal adhesions caused by previous surgeries are responsible for about 75% of cases of SBO; in the United States, about 300 000 patients undergo surgery for bowel obstruction every year due to intra-abdominal adhesions.^2^ A study by Scott et al showed that the prevalence of this etiology did not decrease significantly between 1988 and 2007.^3^ Malignant tumors and hernias are the second and third causes of SBO, respectively.^4^

 The causes of large bowel obstruction (LBO) also vary greatly by geography. Colorectal cancers represent the most common cause of LBO in the United States, while colonic volvulus ranks first in Russia, Eastern Europe, and Africa.^4^ In some countries, including Pakistan, India and Brazil, sigmoid volvulus alone is responsible for 20–30% of cases of LBO, and in Ethiopia, this figure reaches 54%.^5^

 In Iran, among the earliest studies conducted in the field was the one by Prof. Yahya Adl and Dr. Mohammad Alavi, who noted that the most common causes of bowel obstruction in Iran between 1963 and 1967 were hernias, followed by sigmoid volvulus and thirdly, adhesions from previous surgeries.^6^ Also, Vaez-Zadeh et al studied 209 bowel obstruction patients at Shahid Faghihi hospital (Shiraz, Iran) between 1962 and 1967, finding strangulated hernias as the most common cause (41.6%), followed by small bowel volvulus (19.6%), large bowel volvulus (16.7%), and adhesions (14.8%).^7^ Later, Zafarqandi et al found that adhesions were the leading cause of bowel obstruction (23% of all cases) between 1994 and 1996 in Sina Hospital, Tehran, Iran. In the large bowel, volvulus (40%) was the main cause of obstruction, accounting for 9% of all cases, and colorectal cancers (30%) came in second place, accounting for 6% of all bowel obstructions.^8^ In a study conducted by Nahidi and Ghosuri on 214 bowel obstruction patients at Golestan Hospital (Ahvaz, Iran) between 2003–2008, adhesion bands were the most common cause of bowel obstruction (52.4%), followed by stool impaction, ileus, hernia, and colon cancer.^9^ Finally, Akrami et al reviewed the clinical characteristics of 411 patients hospitalized in our center between 2006 and 2012, revealing that the most common intraoperative findings were adhesion band formation (48.1%), masses (17.3%), and hernia (6.7%),^10^ though the study only examined SBO.

 As evident, the etiological characteristics of both small and LBO vary over time. Given that no comprehensive etiological study has been performed in recent years at our center, the present study sought to fill this gap using a very large number of bowel obstruction patients hospitalized in an adult surgery referral center in southern Iran between 2014 and 2020.

## Materials and Methods

 In this cross-sectional study, we retrospectively reviewed the medical records of all patients who were admitted to Shahid Faghihi hospital (Shiraz, Iran) between 2014 and 2020 with a diagnosis of bowel obstruction. After approval of the Ethics Committee of Shiraz University of Medical Sciences, the archives of Shahid Faghihi hospital were searched according to the International Classification of Diseases, Tenth Edition (ICD-10) code of bowel obstruction. Included were all patients hospitalized at our center during the study period with this diagnosis of bowel obstruction according to the ICD-10 code, with no age limitation (N = 2792). The study’s primary measure was the etiology of bowel obstruction, so records with missing or obscure data on this measure were excluded (n = 11). A data collection form was devised to collect the relevant information, including the patients’ age, gender, history of relevant surgeries, comorbidities, cause of obstruction, site of obstruction, type of obstruction, need for surgical intervention, intensive care unit (ICU) admission, length of hospital stay, and final outcome.

 All statistical analyses were made using IBM SPSS version 25.0 (IBM Corp., Armonk, New York, USA). The normality of continuous variables was checked using the Kolmogorov-Smirnov test, revealing a non-normal distribution in all cases. Categorical variables were provided using frequencies and percentages, while continuous variables were reported as the median and interquartile range (IQR).

## Results

 The present study included a total of 2781 patients (61.4% male, 38.6% female) with a median age of 58 (IQR 43–71) years who were admitted to Shahid Faghihi hospital between 2014 and 2020 due to bowel obstruction. The patients ranged from 16 to 94 years of age. Most cases responded to non-surgical treatment (61.3%), while a total of 1081 (38.7%) patients with a median age of 57 (IQR 48–70) years were given surgical treatment, including 683 men (63.2%) and 398 women (36.8%). The obstruction was partial in most cases (65.5%). Overall, 94.4% of cases of complete obstruction required surgery. SBO (72.2%) was almost three times more prevalent than LBO (27.4%). The overall in-hospital mortality rate was 6.2%. Table 1 ranks the etiologies of bowel obstruction according to gender, treatment, type, and outcome.

**Table 1:** Causes of Bowel Obstruction Ranked According to Gender, Type, Treatment, and Overall

**Cause**	**Overall**	**Gender**		**Type**		**Treatment**		**All cases** **(N=2781)**
		**Men** **(n=1713)**	**Female** **(n=1081)**	**Partial** **(n=1830)**	**Complete** **(n=951)**	**Surgery** **(n=1081)**	**Medical** **(n=1680)**	
**Small Bowel Obstruction**								
	n = 2016	n = 1226	n = 790	n = 1529	n = 487	n = 1389	n = 627	
Adhesion bands	66.77	67.54	65.57	76.13	37.37	44.48	76.75	48.40
Carcinomatosis	6.99	5.22	9.75	9.22	‒	3.46	8.64	5.07
Retroperitoneal tumors	6.35	6.04	6.84	7.65	2.26	1.81	8.42	4.60
Inguinal hernia	6.00	5.38	6.96	‒	24.85	18.12	0.79	4.35
Incisional hernia	5.31	7.02	2.66	3.40	11.29	8.90	3.10	3.85
Bezoar	4.22	3.43	5.44	1.44	12.94	12.36	0.72	3.06
Small bowel tumors	2.73	2.67	2.78	‒	11.29	1.81	‒	1.98
Diverticulitis	0.55	0.90	‒	0.72	‒	‒	0.79	0.40
Perforation	0.55	0.90	‒	0.72	‒	9.06	‒	0.40
Fecal impaction	0.55	0.90	‒	0.72	‒	‒	0.79	0.40
**Large Bowel Obstruction**								
	n = 765	n = 487	n = 278	n = 301	n = 464	n = 474	n = 291	
Colorectal tumors	33.90	35.73	30.58	7.31	51.08	47.68	11.34	9.31
Volvulus	29.70	35.73	19.06	‒	48.92	45.78	3.44	8.16
Fecal impaction	21.00	19.92	23.02	53.49	‒	‒	51.89	5.78
Diverticulitis	11.10	8.62	15.47	28.24	‒	4.43	21.99	3.05
Retroperitoneal tumors	4.30	‒	11.87	10.96	‒	2.11	11.34	1.19

All values are percentages within each site (small bowel/large bowel) unless otherwise specified.

**Figure 1:**
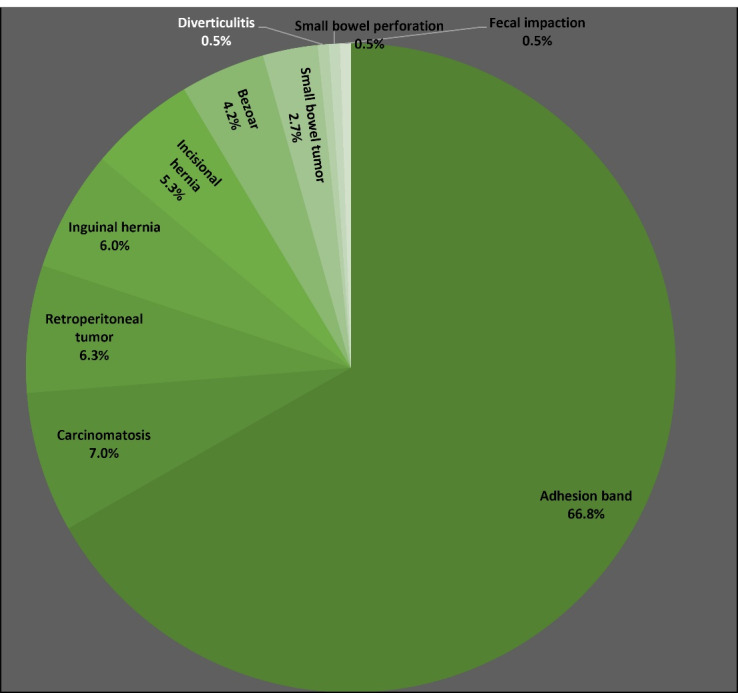
Etiology of small bowel obstruction among 2016 cases at Shahid Faghihi Hospital, Shiraz, Iran, between 2014 and 2020.

Figure 1 summarizes the etiologies of SBO among 2016 cases (60.8% male, 39.2% female) with a median age of 56 (IQR 40–68) years. Most cases (68.9%) were treated non-surgically. Overall, 8.5% required ICU admission for a median length of 3.5 (2.0–6.5) days. The median length of hospitalization was 3 (IQR 1–5) days, and the in-hospital mortality proportion was 3.8% for SBO. Adhesion bands were the most common cause (66.77%), with almost all patients (97.6%) with SBO due to adhesion bands having had previous surgery a median of 24 (IQR 12–60) months earlier, mainly via the open approach (91.5%). The types of previous surgery among those with SBO due to adhesion bands are summarized in Table 2. As evident, colectomy (21.0%) was the most common, followed by stomach or duodenal surgery, appendectomy, small bowel resection, and total abdominal hysterectomy.

**Table 2:** Type of Previous Surgery among 1346 Cases with Small Bowel Obstruction Due to Adhesion Bands

**Previous Surgery**	**N**	**%**
Colectomy	282	20.95
Stomach or duodenal surgery	192	14.26
Appendectomy	172	12.78
Small bowel resection	141	10.48
Total abdominal hysterectomy	116	8.62
Ovarian surgery	88	6.54
Bladder surgery	74	5.50
Open exploration due to trauma	55	4.08
Inguinal hernia	32	2.38
Incisional hernia	32	2.38
Proctectomy	22	1.63
Umbilical hernia	22	1.63
Cholecystectomy	22	1.63
Prostatectomy	21	1.56
Splenectomy	21	1.56
Intussusception surgery	11	0.82
Caesarean section	11	0.82
*No previous surgery*	*32*	2.38
Total	1346	100.00

**Figure 2:**
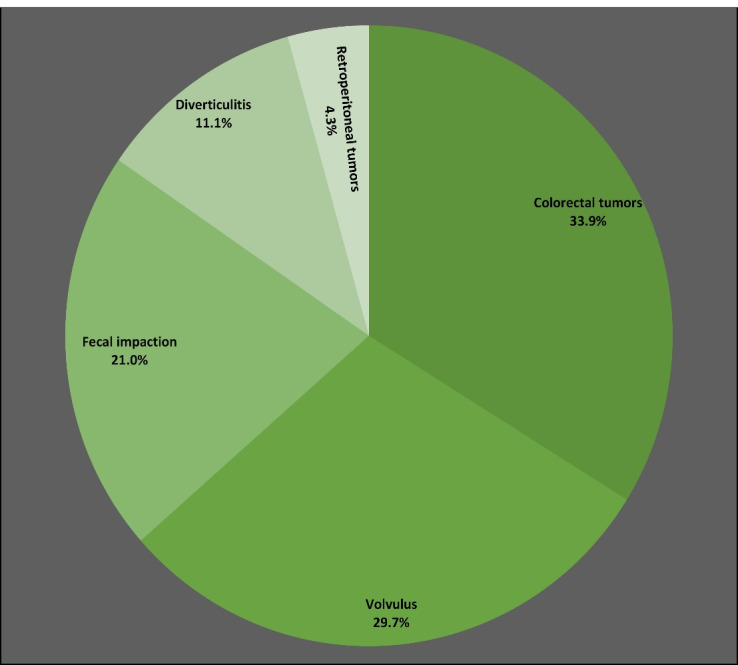
Etiology of large bowel obstruction among 2016 cases at Shahid Faghihi Hospital, Shiraz, Iran, between 2014 and 2020.

 In Figure 2, LBO is classified according to its etiology among 765 cases (63.7% male, 36.3% female). The median age of patients with LBO was 64 (IQR 55–78) years, and the in-hospital mortality proportion was 12.5%. The median length of hospitalization was 4 (IQR 2–8) days. Most patients (62.0%) were treated surgically, representing a higher proportion compared to SBO (30.1%). Almost one-fourth of patients (22.2%) with LBO required admission to the ICU for a median length of 3 (IQR 2–5) days. As seen, the most common causes of LBO were colorectal tumors (33.9%) and volvulus (29.7%).

## Discussion

 The present study reviewed 2781 cases of bowel obstruction managed at Shahid Faghihi hospital (Shiraz, Iran) between 2014 and 2020. Obstruction was mostly encountered among men (61.4%), predominantly in the small intestine (72.2%). The most common causes of small and LBO were adhesion bands and colorectal tumors, respectively. An overall in-hospital mortality proportion of 6.2% was recorded.

 During bowel obstruction, the lumen of the small or large bowel becomes either partially or completely blocked, resulting in abdominal pain, nausea, vomiting, constipation, obstipation, and abdominal distention. As the passage of bowel contents is blocked, a medical emergency may develop, with most patients requiring hospital admission and some requiring surgery.^11^

 Nowadays, the single most common cause of SBO is intra-abdominal adhesions, which are closely associated with a history of prior abdominal surgery.^2^ In our study, two-thirds of all cases of SBO were due to adhesion bands. Similarly, Akrami et al cited adhesion band formation as the most common intraoperative finding (48.1%) among patients with SBO at our center.^10^ Malignant tumors and hernias are the second and third causes of SBO, with our findings agreeing with the literature in this regard.^4,10^ A higher rate of elective hernia repair can explain the decreased rate of SBO due to hernias over time, representing an important advance in preventive measures.

 In our study, most patients with SBO had a history of prior abdominal surgery of the open approach in the preceding two years; colectomy was the most common type of previous surgery, followed by stomach or duodenal surgery, appendectomy, small bowel resection, and total abdominal hysterectomy. Methods to prevent the postoperative complication of adhesion band formation include a shift toward non-operative management where possible and laparoscopic rather than open surgery if operative management is indicated.^12^ Barrier agents have also been investigated, though a recent retrospective cohort study of 57 499 patients in Japan revealed that they did not show clear effectiveness for the prevention of bowel obstruction after laparoscopic surgery.^13^ Adjunct treatments have also been considered for adhesive SBO, with olive oil shown to decrease the hospital stay and spontaneous resolution time in selected cases when combined with standard treatment.^14^ Finally, Tang et al expressed the need to develop safer and more effective anti-adhesion agents by understanding the pathological process of adhesion formation and maintaining the balance of multiple feedback systems (coagulation, inflammation, fibrinolysis and related cells, molecules, and regulatory signals) involved in injury repair.^12^

 LBO accounts for roughly 10–25% of all cases of bowel obstruction, with colorectal adenocarcinoma (50–60%), diverticular disease (10–20%), and volvulus (10–15%) accounting for most cases in the United States.^11,15^ In the present study, the most common causes of LBO were colorectal tumors (33.9%) and volvulus (29.7%), with fecal impaction (21.0%) and diverticulitis (11.1%) ranking third and fourth. These findings can be explained by the lower rate of colorectal cancer in Iran relative to Western countries.^16^ However, we should not neglect the fact that the incidence of colorectal cancer has been rising in recent decades in Iran,^16^ with the city of Shiraz being no exception.^17^ In fact, cancer was not among the prominent etiologies of bowel obstruction in the studies conducted in Iran between 1963 and 1967,^6,7^ though it emerged as an important cause in the 1994–1996 study by Zafarqandi et al.^8^ This phenomenon may be due to changes in individual and environmental risk factors, improved registry systems, increased access to health services, and an aging population.^16^ It is also essential to consider that males and females are at risk of colorectal cancer at younger ages in Middle-Eastern countries compared to Western countries.^18^ Hence, policymakers should improve cancer surveillance programs for earlier detection of colorectal cancer in the Iranian population.

 Concerning the gender of adult bowel obstruction patients, some reviews have suggested that the incidence of both small and LBO is similar across the genders.^11^ However, males comprised the majority of our cases for SBO (60.8%), LBO (63.7%), and overall (61.4%). Our findings are in line with those from a 2003–2008 study in Ahwaz, Iran, where 60.3% of patients with bowel obstruction were male^9^; the 2006–2012 study at our center also revealed a similar proportion of males (61.5%).^10^ However, the 1994–1996 study of Zafarqandi et al in Tehran, Iran, showed a stronger (70%) male predominance, while the 1963–1967 study of Adl and Alavi revealed an even higher proportion of male patients (78.3%).^6^ It seems reasonable to conclude that men are at a higher risk of bowel obstruction in Iran, probably due to lifestyle habits.

 At the same time, there seems to be an increase in the proportion of women being affected by bowel obstruction in Iran over time, which may be associated with the parallel rise in the trend toward Cesarean sections in recent decades.^19^ In fact, a UK cohort of 81 460 women revealed that the risk of SBO was higher among women with a history of a Cesarean section compared to vaginal delivery.^20^ In Poland, one single-center study looked at changes in mechanical bowel obstruction over the past 145 years and found a rise in the number of women affected by this condition, together with increased cancer and adhesion cases over time.^21^ Hence, the gender discrepancy related to bowel obstruction appears to be fading gradually, and modifiable risk factors should be determined in order to guide much-needed preventive interventions.

 In the present study, the median age of patients with SBO was 56 (IQR 40–68) years, compared with 64 (IQR 55–78) years among those with LBO. These values are higher than those recorded in earlier Iranian studies,^6-8,10^ probably reflecting increased life expectancy and population aging. A 2011 study in Nigeria recorded a median age of just 49 years among 50 patients with LBO, though the average life expectancy was just 51.35 years.^22^ The 145-year retrospective Polish study also noted an increase in the mean age of patients with mechanical bowel obstruction over time.^21^ It seems that as life expectancy increases and people are exposed to more triggering factors, bowel obstruction affects people in higher age groups, with poorer outcomes eventuating. As such, studies focusing on bowel obstruction prevention and management in the geriatric population are necessary.

 According to the Bologna guidelines, most patients with an acute obstruction of the small bowel do not require surgical treatment; this was highlighted by our results, where 68.9% of such cases were managed non-operatively. Similarly, the previous study at our center reported that 73.6% of patients were managed conservatively.^10^ Of course, surgery is indicated when there are signs of peritonitis, strangulation, or ischemia, which is why roughly a third of cases did undergo surgery.^23^ In a 1998–2002 study of 32 583 patients with a SBO in California, those who underwent operations during the index admission had longer lengths of stay, lower mortality, fewer SBO readmissions, and longer time to readmission than patients treated non-surgically.^24^ The recurrence of SBO is highly dependent on the number of previous episodes, with one study finding lower recurrence in those managed operatively and recommending operative management after the first recurrence.^25^ In a systematic review of 38 057 patients with adhesional SBO treated surgically, laparoscopic adhesiolysis was linked to decreased morbidity and mortality risk than the open approach.^26^ Springer et al prospectively studied 104 elderly patients (mean age 79 years, 43% male) with SBO in Canada. While surgery led to more complications and a longer hospital stay, non-operative management was linked with a high recurrence rate. However, surgery after failed non-operative management was associated with increased mortality than immediate surgery, suggesting that some elderly patients may be waiting too long for surgery.^27^

 On the other hand, LBO often requires surgical management due to its etiologies. Surgical management is indicated after initial supportive therapy (fluid resuscitation, electrolyte correction, etc) in patients with malignant obstruction, volvulus refractory to non-surgical decompression, perforation, ischemia, or fecal peritonitis.^3,28^ In line with the literature, most cases of LBO (62.0%) required surgery in our study. It should be noted that for cases with colonic cancer, laparoscopic surgery has been linked with a lower risk of subsequent SBO and less associated mortality than open surgery.^29^

 Our study faced several limitations. Firstly, late postoperative complications could not be assessed due to the study’s retrospective nature and the lack of follow-up on patients after discharge. Secondly, our investigation was cross-sectional, so although a much larger number of cases were found than in the previous study (perhaps explained by the longer study period, the inclusion of both small and LBO, and possibly improved patient registration),^10^ we could not conclude that there has been an increase in incidence. Hence, longitudinal studies are essential. Finally, data on the clinical presentation and laboratory parameters were not collected. In light of these limitations, we propose that a database be designed to collect data on patients with bowel obstruction prospectively.

 In conclusion,this study reviewed 2781 cases and found adhesion bands and colorectal tumors were the most common causes of adult small and LBO, respectively. Alarmingly, colorectal and retroperitoneal tumors accounted for almost half the mortalities. The predominant etiology of LBO was colorectal cancer, suggesting that policymakers should improve surveillance programs to detect the condition earlier in the aging Iranian population. Regarding SBO, the leading cause was adhesion bands, indicating the necessity of further efforts to reduce the rate of adhesions following intra-abdominal operations.
